# Numerical Analysis of Laser-Excited SAM-Coated Magnetic Nanoparticles for Electromagnetic Field Enhancement in Optical Gas Sensing

**DOI:** 10.3390/s26010031

**Published:** 2025-12-20

**Authors:** Jong Hyun Kim, Hae Woon Choi

**Affiliations:** Department of Mechanical Engineering, Keimyung University, Daegu 42601, Republic of Korea; kimjh@kmu.ac.kr

**Keywords:** particle sensing, SAM, laser irradiation, laser beam scattering

## Abstract

This study investigates the electromagnetic field enhancement and optical response of self-assembled monolayer (SAM)-coated iron nanoparticles under laser excitation, with the aim of advancing optical gas sensing technologies. Using finite element method (FEM) simulations, we model the interaction of laser beams in both the visible (400–700 nm) and infrared (1000–2500 nm) spectral ranges with SAM-coated and uncoated nanoparticles. The results reveal that SAM coatings significantly amplify localized electromagnetic fields—reaching up to ~60 V/m in the visible range—while providing stable, wavelength-independent field distributions. In contrast, uncoated nanoparticles exhibit weaker but more variable field responses. Angular dependence analysis indicates maximal field enhancement at perpendicular (90°) detection, suggesting an orientation-sensitive design consideration for optical sensors. These findings demonstrate that SAM coatings enable stable, wavelength-independent electromagnetic responses, offering a promising pathway toward miniaturized and highly sensitive laser-based optical gas sensors.

## 1. Introduction

Laser-based optical gas sensors offer high sensitivity, selectivity, and rapid response for environmental and industrial monitoring. Enhancing their performance increasingly relies on nanostructured materials that can amplify local electromagnetic fields under laser illumination. Amid mounting global concerns surrounding environmental sustainability, energy efficiency, and ecological balance, the development of advanced gas sensing technologies has become increasingly vital across environmental, biomedical, and industrial domains. Among the available options, optical gas sensors—particularly those based on laser spectroscopy—have attracted significant interest owing to their high sensitivity, molecular selectivity, rapid response, and non-invasive nature. To further enhance detection performance, researchers are increasingly integrating nanomaterials with engineered surface functionalities into these optical platforms [1,2,3,4,5,6].

To characterize gaseous materials, laser beam scattering can be used. Scattering analysis enables the optimization of nanoparticle size, morphology, and surface chemistry to maximize electromagnetic field enhancement—an effect that significantly amplifies optical signals such as Raman scattering, fluorescence, or absorption. This enhancement is particularly valuable for surface-enhanced Raman spectroscopy (SERS)-based gas sensing, where the intensity of the Raman signal scales with the local electric field near the nanoparticle surface. By understanding the scattering cross-section and angular distribution under various laser excitation conditions, researchers can tailor nanoparticle synthesis (e.g., through laser ablation parameters) to achieve maximum sensitivity and selectivity of gaseous materials. Ultimately, laser beam scattering analysis bridges the gap between material design and sensor performance, guiding the rational development of highly sensitive, miniaturized, and real-time optical gas sensing platforms for environmental monitoring, industrial safety, and biomedical diagnostics [7,8,9].

Among such materials, magnetic nanoparticles (MNPs) coated with self-assembled monolayers (SAMs) are promising because SAMs provide both surface stability and specific molecular recognition sites for gas adsorption. Under laser excitation, these coatings also modify the local dielectric environment, influencing electromagnetic confinement and light–matter interactions critical for optical sensing [7,9,10]. One promising approach involves the use of self-assembled monolayers (SAMs)—highly ordered molecular films that form spontaneously on solid surfaces—as surface functionalization agents for nanoparticles. When immobilized on magnetic nanoparticles such as iron oxide, SAMs not only prevent particle agglomeration and improve colloidal stability but also introduce selective chemical binding sites for gas molecules [7,10]. This interface engineering plays a critical role in enhancing local electric fields under laser excitation, thereby modulating light–matter interactions central to the generation of measurable optical signals. However, quantitative understanding of how SAM coatings affect near-field distribution and scattering in magnetic nanoparticles remains limited. Analytical Mie theory cannot easily capture multilayer and orientation effects, especially across the visible–infrared range [7,8,9,10].

Magnetic nanoparticles (MNPs) have emerged as powerful platforms for advanced functional materials, extending beyond biomedical applications to next-generation gas sensing technologies. As Fe and Fe-oxide magnetic nanoparticles are widely used as robust, low-cost cores that can be synthesized via pulsed laser ablation or solution routes and easily coated with SAMs or silica/organic shells; prior studies have shown that such particles provide good stability and tunable surface chemistry for sensing applications. In industrial/environmental applications, researchers study the effects of magnetic fields on particles suspended in gas streams (e.g., fly ash, air pollutants) to improve filtering and separation efficiency in industrial settings. Gas-Assisted Liquid Separation (GALS) is a technique whereby gas bubbles are introduced into a liquid suspension to aid in the recovery of protein-loaded magnetic particles, which helps with large-scale processing.

Fe-based magnetic nanoparticles coated with organic SAMs combine a robust, magnetically controllable core with a chemically selective surface layer. Under laser illumination, the SAM region provides adsorption sites for gas molecules and a tailored dielectric environment in which local electromagnetic fields can be enhanced and directed. This study therefore examines how SAM functionalization on Fe nanoparticles modifies near-field enhancement and angular scattering, in order to evaluate whether laser beam scattering can distinguish between bare and SAM-coated particles under identical conditions.

When coated with self-assembled monolayers (SAMs) or aerosol—highly ordered molecular films spontaneously formed on the nanoparticle surface—MNPs acquire tunable surface chemistry capable of selectively capturing or interacting with target gas molecules. This molecular-level functionalization enables not only selective gas adsorption but also enhanced optical response through localized electromagnetic field amplification under laser excitation [7,8,9,10]. However, precise characterization of these SAM-coated nanoparticles is challenging due to their nanoscale dimensions and polydispersity. To overcome these challenges, Rivera-Chaverra et al. synthesized iron oxide nanoparticles via pulsed laser ablation and characterized their size and morphology using dynamic light scattering (DLS) and scanning transmission electron microscopy (STEM) [10]. Their study demonstrated that laser energy strongly affects particle size distribution and scattering behavior—parameters that directly influence the localized field enhancement crucial for optical gas sensing. These insights highlight the potential of laser-engineered, SAM-functionalized magnetic nanoparticles as sensitive, selective platforms for next-generation optical gas detection systems [7,8,9,11,12,13].

Santillan et al. investigated the optical properties of Fe nanoparticles synthesized via ultrashort pulsed laser ablation, emphasizing how particle size and morphology govern light–nanoparticle interactions, including scattering behavior [7]. This is highly relevant for gas sensing, where controlled scattering can amplify optical signals associated with gas adsorption events. For further characterization, Ndukwe-Ajala et al. examined the scattering response of ultrafine γ-Fe_2_O_3_ nanoparticles under applied magnetic fields, demonstrating that magnetic fields can dynamically tune scattering intensity and angular distribution [11,12]. Such magneto-optical control could be exploited to modulate sensor response or enhance detection specificity for paramagnetic gaseous species [9,11,12]. Complementary to these findings, Gogoi et al. reported that nanoparticles act as efficient scattering and absorption centers under near-infrared irradiation, redistributing energy and locally enhancing electromagnetic fields [12]. This localized field enhancement is particularly valuable for optical gas sensors based on Raman or infrared spectroscopy, where higher field intensity translates into stronger, more detectable signals even at trace gas concentrations [7,8,9]. Together, these studies underscore the importance of laser-induced scattering analysis for designing SAM-functionalized magnetic nanoparticles optimized for highly sensitive and tunable gas detection platforms [7,8,9].

As a gaseous material detection example, CuFe_2_O_4_ nanoparticles synthesized via green routes exhibit multifunctional capabilities, including effective sensing of hydrogen sulfide (H_2_S), owing to their electrochemical activity and surface reactivity [13]. Meanwhile, rare-earth-doped materials, such as Er^3+^:GdOF nanoparticles, have demonstrated high thermal sensitivity and spatial resolution in optical nanothermometry, making them promising for in-situ temperature monitoring and thermally coupled sensing platforms [14]. The combination of optical sensitivity and thermal responsiveness enables the design of hybrid sensors capable of detecting both chemical and thermal signatures of target analytes [13,14].

Furthermore, tuning the parameters of laser excitation—such as pulse energy and wavelength—can significantly enhance signal intensity in laser-induced breakdown spectroscopy (LIBS), thereby improving detection limits for trace analytes [15,16]. For field applications, standoff LIBS systems have also proven effective for remote, real-time analysis of gaseous or aerosolized pollutants in ambient air [1,5]. Collectively, these advancements point toward a next generation of smart, miniaturized, and eco-friendly optical gas sensors that synergistically integrate surface-engineered nanomaterials, laser modulation strategies, and remote sensing capabilities [2,3,4,5,6].

The primary objective of this study is to investigate the laser beam–induced scattering behavior and electric field response of iron nanoparticles and SAM (Self-Assembled Monolayer)-coated iron colloids, with an emphasis on their relevance to gas sensing applications. Particular attention is given to understanding how the presence of the SAM layer modifies the optical and electromagnetic properties of the nanoparticles across different spectral regions, including the visible and infrared (IR) ranges. These modifications directly affect light scattering intensity and spatial field distribution—key parameters that determine the sensitivity of optical gas detection techniques. To elucidate these effects, both simulation models and experimental measurements are employed, providing a comprehensive analysis of electric field localization, scattering cross-sections, and intensity enhancement under laser excitation. The resulting data reveal how SAM functionalization can be exploited to optimize scattering-based signal amplification, thereby enabling the design of highly sensitive nanoparticle-based gas sensors [7,8,9,10,11].

Using finite element method (FEM) simulations, we analyze how SAM functionalization modifies the localized electric field distribution and scattering intensity around the nanoparticles. The results reveal that the SAM layer not only alters near-field enhancement but also improves the stability and reproducibility of the optical response under laser illumination—factors critical for reliable gas detection. These findings demonstrate the potential of SAM-coated iron nanoparticles as engineered scattering centers for laser-based gas sensors, enabling enhanced light–matter interaction and improved detection sensitivity for trace gaseous analytes [7,8,9,10,11].

As described in previous section, laser beam scattering analysis provides a powerful, non-destructive, and highly sensitive approach for characterizing colloidal particles. It enables real-time monitoring of particle size, distribution, and dynamics without altering their chemical or structural integrity, making it ideal for studying reactive or unstable suspensions. Because the scattered light intensity and angular distribution depend on the particle’s size, refractive index, and morphology, this technique allows precise determination of particle size distributions across the nanometer-to-micrometer range using models such as Mie and Rayleigh scattering. Additionally, variations in scattering spectra reveal valuable information about surface coatings, dielectric environments, and interfacial properties—such as the presence and thickness of self-assembled monolayers (SAMs)—that influence optical and electromagnetic behavior. Laser scattering also provides insight into localized surface plasmon resonance (LSPR) and Mie resonance phenomena, which are central to understanding light–matter interactions in metallic and magnetic colloids [8,9]. Furthermore, it integrates seamlessly with numerical simulations like finite element or finite-difference time-domain (FDTD) methods, enabling comprehensive analysis of near-field enhancement and scattering cross-sections. Overall, laser beam scattering serves as a bridge between optical diagnostics and material science, offering a versatile and precise means to probe the structural, chemical, and electromagnetic characteristics of colloidal nanoparticles for applications in sensing, photonics, and nanotechnology [7,8,9,10,17,18,19,20].

This study employs finite element method (FEM) simulations to analyze the laser–nanoparticle interaction in SAM-coated versus uncoated Fe nanoparticles. The work aims to reveal how SAM coatings influence field localization, wavelength dependence, and angular enhancement—parameters directly related to optical signal amplification mechanisms such as Raman or infrared absorption in gas detection. The purpose of this paper is to investigate the electromagnetic field enhancement and optical response of self-assembled monolayer (SAM)-coated iron nanoparticles under laser excitation, with the ultimate goal of advancing laser-based gas sensing technologies. By employing finite element method (FEM) simulations, the study aims to elucidate how SAM coatings influence the interaction between incident laser beams and magnetic nanoparticles across both visible and infrared spectral regions. Specifically, the paper focuses on analyzing changes in scattering intensity, localized electric field distribution, and angular dependence to understand how surface functionalization alters light–matter interactions at the nanoscale. Through these analyses, the research seeks to establish SAM-coated nanoparticles as controllable and efficient scattering centers, capable of enhancing detection sensitivity and stability in optical gas sensors. Ultimately, the study provides a theoretical and computational foundation for designing next-generation miniaturized, highly sensitive, and wavelength-independent laser sensing systems [8,9,21,22].

The results provide a theoretical foundation for designing stable, wavelength-independent, and orientation-sensitive optical gas sensors. Section 2 introduces the theoretical background of laser scattering and core–shell modeling, Section 3 describes the FEM approach and material parameters, and Section 4 discusses field enhancement behavior and its implications for gas sensing.

## 2. Theoretical Background

### 2.1. Theory of Laser Beam Scattering for Nanoparticles

Laser beam scattering by nanoparticles is governed by the interaction of incident electromagnetic radiation with particles whose dimensions are comparable to or smaller than the wavelength of the light [21,22,23,24,25,26,27,28]. The fundamental theoretical framework for describing such scattering includes Mie theory and Rayleigh scattering, depending on the particle size parameter (x = 2πr/λ). Here, r represents the nanoparticle radius, and λ denotes the incident laser wavelength. For particles much smaller than the wavelength (x ≪ 1), Rayleigh scattering dominates, leading to strong wavelength dependence and angular distribution of scattered intensity. For particles with a size comparable to the wavelength (x~1), Mie theory is used, accounting for complex multipole interactions and interference effects [26,28].

The scattering intensity depends on the incident wavelength, refractive index contrast between the particle and the medium, and the geometry of the particle. In the case of metallic or magnetic nanoparticles, localized surface plasmon resonances (LSPRs) can arise, where conduction electrons collectively oscillate in response to the electric field of the laser. These resonances enhance the local electric field near the nanoparticle surface and significantly amplify scattering and absorption cross-sections. The presence of a SAM layer alters the effective dielectric environment, leading to a shift in resonance conditions and field distribution [7,8,9,10].

The scattered electric field can be described as a function of the dipole moment induced in the nanoparticle by the incident laser field, which itself is influenced by the shape, composition, and surrounding dielectric properties. For spherical nanoparticles, the polarizability is given by

(1) $$\alpha =4\pi \epsilon_{0}r^{3}\left(\frac{\epsilon_{p}-\epsilon_{m}}{\epsilon_{p}+2\epsilon_{m}}\right)$$

where $\epsilon_{p}$ is the complex permittivity of the particle, $\epsilon_{m}$ is the permittivity of the surrounding medium, and r is the particle radius. This polarizability governs the magnitude of induced dipole scattering and is modified by the presence of surface coatings and complex geometries.

In practical applications, numerical methods such as the finite element method (FEM), finite-difference time-domain (FDTD) and discrete dipole approximation (DDA) are often employed to simulate near-field and far-field scattering patterns. These approaches account for real particle geometries, multilayer coatings (e.g., SAMs), and substrate effects, providing accurate predictions of field enhancement and energy dissipation critical for sensor performance [8,9,21,22,23,24,25].

Laser-induced nanoparticle synthesis and scattering analysis have gained increasing attention for their relevance in both optical diagnostics and materials design. The pulsed laser ablation (PLA) technique was demonstrated in liquid for synthesizing scheelite-type nanoparticles such as CaWO_4_ and CaMoO_4_, showing that the produced particles were spherical with narrow size distributions (16–29 nm) and well-dispersed in colloidal form [19]. To analyze scattering behavior and size distributions, they employed nanoparticle tracking analysis (NTA), which utilized Brownian motion data extracted from optical microscopy to resolve size distribution functions. This method, sensitive to particle dynamics and optical scattering, enabled real-time visualization and quantification of sub-100 nm particles, a critical regime where Rayleigh and Mie scattering transition [19,20].

In a complementary study, nanocalorimetry was studied to investigate phase transitions in metallic nanoparticles, indirectly contributing to the understanding of optical scattering by capturing size-dependent melting behaviors [10]. Their high-speed thermal analysis provided insights into how size, crystallinity, and nucleation pathways influence nanoparticle phase behavior, which correlates with changes in dielectric properties and, by extension, light scattering profiles. The calorimetric measurements, capable of capturing rapid thermal transitions with sub-nanogram sensitivity, offered a framework to validate simulation models that incorporate thermodynamic parameters into optical scattering calculations [20]. Together, these works illustrate how experimental simulation and analysis techniques can jointly inform the modeling of nanoparticle scattering, supporting accurate prediction of resonance conditions and optical field distributions in engineered nanomaterials [19,20,21,22,23,24,25].

### 2.2. Nanoparticle-Enhanced Scattering for Gas Sensing

Recent studies have emphasized the importance of nanoparticle morphology, composition, and dispersion in modulating light–matter interactions under laser excitation [8,13,14,22,23,24]. For instance, the dispersion quality of carbon black nanoparticles (CBNs) in self-sensing cementitious composites was shown to be critically dependent on sonication energy, with dynamic light scattering (DLS) and zeta potential analyses revealing optimal dispersion and enhanced electromechanical responses at ~240 J/mL [10]. Similarly, CuFe_2_O_4_ ferrite nanoparticles synthesized via green routes exhibited efficient electron transfer and electromagnetic coupling, facilitating photocatalytic and sensing functions due to their tunable spinel structures and high surface area [13]. Furthermore, the optical and plasmonic scattering behavior of bimetallic Au–Ag nanoparticles has been leveraged for formaldehyde sensing and imaging due to their shape-dependent field enhancement and resonant absorption properties [24]. These findings underscore the role of nanoparticle design and surface engineering in governing electromagnetic field interactions, making them crucial for developing next-generation laser-driven gas sensors [8,13,14,23,24].

Laser-based spectroscopy techniques continue to evolve, offering powerful platforms for quantitative gas detection, trace analysis, and nanoparticle diagnostics. For example, Laser-Induced Breakdown Spectroscopy (LIBS) has been demonstrated as a rapid, multi-elemental analytical method for detecting trace elements in both environmental and biological samples [22,23,24,25]. The technique has found applications in surface analysis of battery materials and elemental imaging of complex matrices such as food products and biological tissues. Meanwhile, photoacoustic and infrared laser absorption methods have enabled precise gas quantification even in mixed or low-concentration environments [3,6].

The value of laser spectroscopy in combustion diagnostics and nanomaterial applications was demonstrated [26,27,28]. A non-invasive flame diagnostic method combined two-color thermometry and optical flow algorithms to simultaneously capture flame temperature and velocity fields using a single camera, thereby simplifying sensor design and reducing costs [26]. Laser-induced ignition of boron-based nano-enhanced fuels found that Bi_2_O_3_ and CuO additives significantly reduced ignition temperature and enhanced combustion rates due to better heat absorption and plasma generation under laser irradiation [15,28]. Additionally, an experimental investigation carried out on particulate behavior in combustion environments and highlighted how laser-based diagnostics can elucidate fine particle evolution in partial-flow diesel filters [28]. These findings collectively underscore the importance of precise laser–matter interaction studies for developing high-performance sensors and advanced energetic materials [15,26,27,28].

Furthermore, advancements in combustion diagnostics and emission control have increasingly leveraged laser spectroscopy and AI-driven modeling [27,28]. For instance, convolutional neural networks (CNNs) and long short-term memory (LSTM) models were used to optimize potassium-induced slag detection in biomass combustion via flame image classification, achieving over 98% accuracy using enhanced GoogleNet models [27]. Particle formation during the combustion of raw biomass and its pyrolysis products was studied, highlighting the dramatic reduction in fly ash emissions when using bio-oils as low-ash fuels, which is advantageous for optical detection in clean environments [28]. These studies underscore the synergy between optical diagnostics, advanced data analysis, and materials science in the pursuit of cleaner combustion and more sensitive gas detection.

## 3. Computational Analysis

### Numerical Modeling

**Figure 1:**
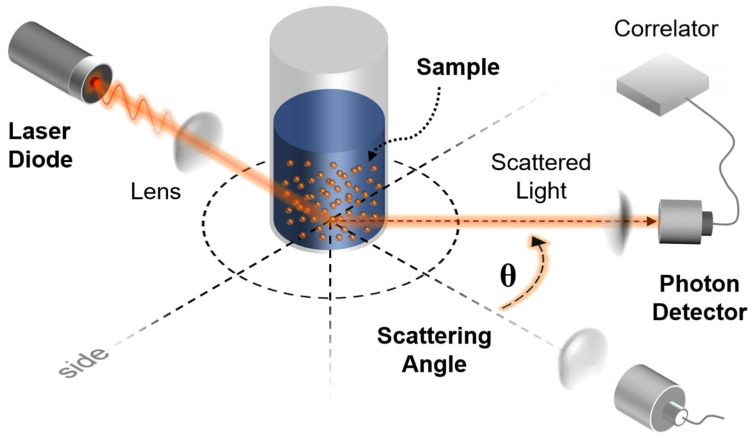
Laser beam irradiation and scattering model. The waves of laser beam is generated through the diode, and penetrated through lenses. Nanoparticles in the solution scatters the pathway of the waves. Then the phonon detector recognizes the angle of scattered light.

The fundamental governing equation, Maxwell’s equation for time-harmonic electromagnetic fields which lead to the vector Helmholtz equation can be approximated using Mie scattering theory [26,28]. In this study, iron nanoparticles with a radius of 100 nm and a self-assembled monolayer (SAM) coating of 30 nm thickness were analyzed as a model for drug delivery applications in magnetic resonance [23,24,25]. To determine an appropriate coating thickness, a preliminary parametric analysis was conducted for shell thicknesses between 5 nm and 50 nm. The normalized field enhancement increased rapidly up to approximately 25–35 nm and then reached a plateau, indicating that beyond this range, additional thickness contributed little to further confinement. A value of 30 nm was therefore selected as a representative thickness that maximizes enhancement while ensuring mesh stability. This value also corresponds to the experimentally observed range of effective dielectric coatings for SAM-grafted or silane-based organic layers (10–50 nm) [23,24,25,26]. Prior reports on iron-oxide nanoparticles are cited as related background, but our simulations use Fe cores [23,24,25]. The excitation wavelengths of the laser beam were selected as 400–700 nm in the visible spectrum and 1000–2500 nm in the infrared spectrum (Figure 1).

**Figure 2:**
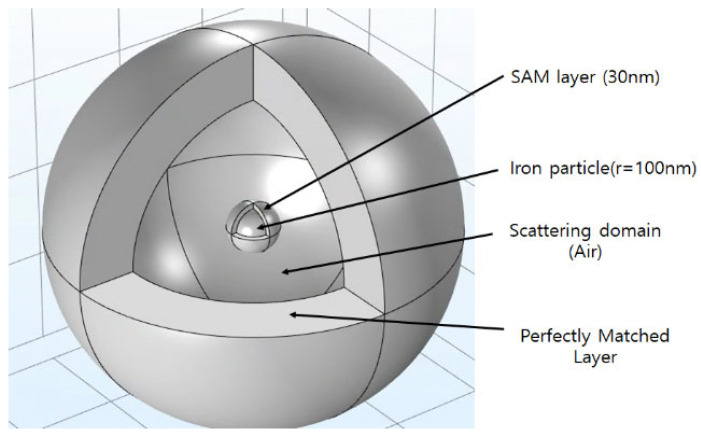
Simulation model. She SAM/Fe core–shell nanoparticles analyzed in this work have been with Air boundary momain.

Finite Element Method (FEM) simulations were conducted using COMSOL Multiphysics (version 5.2), employing the Wave Optics module for numerical analysis [19,22,26,28]. The computational domain in the simulation consists of three regions. Figure 2 presents the finite element simulation model constructed in COMSOL Multiphysics to analyze how a laser beam interacts with a self-assembled monolayer (SAM)–coated iron nanoparticle [19,26]. The model consists of three key domains: the central particle region, a surrounding scattering region, and an outer perfectly matched layer (PML).

In the FEM model, a Perfectly Matched Layer (PML) was implemented at the outer boundary of the computational domain to eliminate artificial reflections of scattered electromagnetic waves. The PML is not a physical layer but a numerical absorbing boundary that matches the electromagnetic impedance of the surrounding medium—air in this case—and exponentially attenuates outgoing waves. This ensures that all scattered fields exit the simulation domain as if propagating into an infinite free-space environment, thereby accurately reproducing real optical conditions around the nanoparticle.

At the core, a 100 nm iron nanoparticle is enveloped by a 30 nm-thick SAM coating that modifies its local dielectric environment and surface charge distribution. The scattering region represents the surrounding medium—air or liquid—through which the plane-wave laser propagates and interacts with the nanoparticle. Within this zone, the near-field and far-field electromagnetic responses are computed to determine how coating thickness and wavelength affect field intensity and scattering patterns. To prevent artificial reflections from the model boundaries, the outermost PML absorbs outgoing electromagnetic waves, effectively simulating an infinite optical domain. This configuration allows accurate calculation of electric-field distributions, resonance behavior, and wavelength-dependent scattering characteristics, thereby providing a robust numerical framework for comparing the optical responses of SAM-coated and uncoated nanoparticles under laser excitation [22,23,24,25,26,28].

The optical constants used in the FEM simulations are summarized in Table 1. For Fe, the complex refractive index was taken from tabulated literature data and implemented as a wavelength-dependent dispersive material in the simulation software, with n = 2.85 + 3.15i at 550 nm reported in Table 1 as a representative value. The SAM coating was modeled as a typical alkyl-silane (octadecyl-trichlorosilane, OTS) layer with n ≈ 1.45, corresponding to a real permittivity ε_r_ ≈ 2.1, representative of common organic self-assembled monolayers whose refractive index varies only weakly over the 400–2500 nm range. The surrounding medium was assumed to be air (n = 1.0003). Within this Fe/SAM-OTS/air configuration, the weak dispersion of the SAM layer was found to have a negligible influence on the calculated field-enhancement trends, so the reported results should be regarded as representative rather than universal for all SAM materials [26]. Owing to the small thickness of the SAM layer (30 nm), additional modulation of the optical response by the residual wavelength dependence of its refractive index was neglected in the paper.

In the simulation, the plane wave applied was of the form of the simple Equation (2)

(2) $$E(x,t)=E_{0}cos\left(kx-\omega t+\emptyset \right)$$

where E_0_ denotes the incident plane-wave amplitude of the background field used to illuminate the particle, E(x,t): the electric field at a specific position (x) and time (t), E_0_: the amplitude of the wave, which is the maximum strength of the electric field, k: the wave number, ω: angular frequency. The laser beam that is not absorbed by the particles scatters, and the scattering behavior depends on the size of the particles and the applied wavelength. In general, when the size of the particle and the wavelength applied are similar, Mie scattering theory is used to predict the scattering behavior, which is predictable when the laser beam is applied to nanoscale particles, as shown in Equations (3)–(5) [26,28]. Equations (3)–(5) summarize the classical Mie solution for a single homogeneous sphere and are included here only as a theoretical reference for understanding size-parameter and multipole effects; the SAM/Fe core–shell nanoparticles analyzed in this work are instead modeled by directly solving Maxwell’s equations in COMSOL’s Wave Optics module for the full core–shell geometry shown in Figure 2. The scattered field is expressed as a sum of spherical Bessel and Hankel functions:

(3) $$E_{sca}=\sum_{n=1}^{\infty }(a_{n}N_{n}^{\left(3\right)}+b_{n}M_{n}^{\left(3\right)})$$

(4) $$a_{n}=\frac{m\varphi_{n}\left(mx\right){\varphi^{\prime }}_{n}\left(x\right)-\varphi_{n}\left(x\right){\varphi^{\prime }}_{n}\left(mx\right)}{m\varphi_{n}\left(mx\right){\zeta^{\prime }}_{n}\left(x\right)-\zeta_{n}(x){\varphi^{\prime }}_{n}\left(mx\right)}$$

(5) $$b_{n}=\frac{\varphi_{n}\left(mx\right){\varphi^{\prime }}_{n}\left(x\right)-m\varphi_{n}\left(x\right){\varphi^{\prime }}_{n}\left(mx\right)}{\varphi_{n}\left(mx\right){\zeta^{\prime }}_{n}\left(x\right)-{m\zeta }_{n}(x){\varphi^{\prime }}_{n}\left(mx\right)}$$

where $M_{n}^{\left(3\right)}$, $N_{n}^{\left(3\right)}$ are vector spherical harmonics, $a_{n}$ and $b_{n}$ are the Mie coefficients, which describe the strength of electric and magnetic multi-poles. Size parameter $x=\frac{2\pi r}{\lambda }$, $m=\frac{n_{p}}{n_{m}}$ is the relative refractive index (particle to medium). $\varphi_{n}$ and $\zeta_{n}$ are Riccati-Bessel and Hankel functions. For layered nanoparticles such as SAM-coated structures, the standard Mie scattering equations are extended to include additional dielectric interfaces. The boundary conditions for electric and magnetic fields are applied at both the core–shell and shell–medium boundaries, leading to recursive expressions for the Mie coefficients. In this study, these multilayer effects were implemented in COMSOL Multiphysics through direct numerical solution of Maxwell’s equations for the core–shell geometry [26,28,29,30,31,32].

## 4. Results

**Figure 3:**
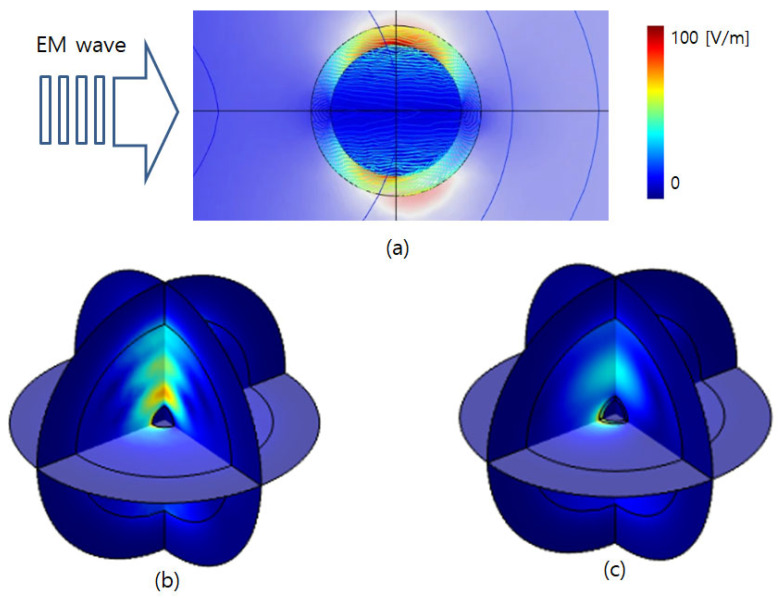
Electric field response for (**a**) excitation direction, (**b**) a nanoparticle with an SAM layer, and (**c**) a nanoparticle.

**Figure 4:**
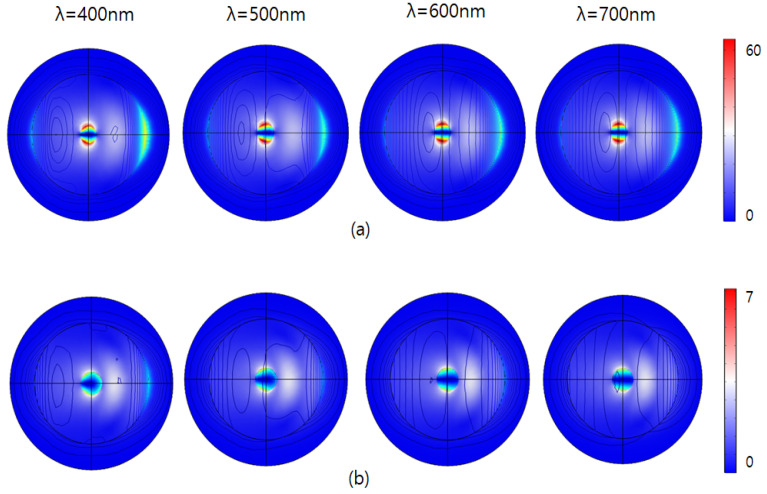
E-field intensity |E|/|E_0_| by visible range laser for (**a**) with an SAM (**b**) without an SAM.

**Figure 5:**
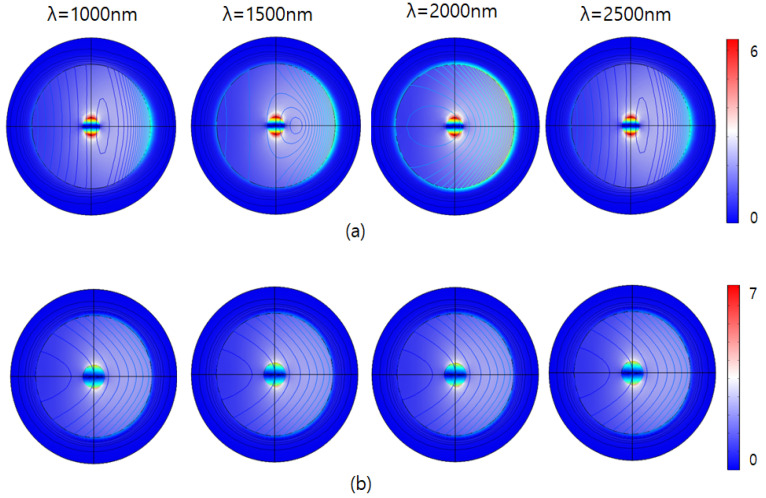
E-field intensity (|E|/|E_0_|) by IR range laser (**a**) with an SAM (**b**) without an SAM.

To enable direct comparison, we report both absolute fields (V/m) and the dimensionless enhancement |E|/|E_0_|. With E_0_ = 1 V/m, the color scales in Figure 3, Figure 4 and Figure 5 are numerically equal to |E|/|E_0_|. Figure 3 illustrates the excitation of nanoparticles by an incident laser beam and the resulting electromagnetic field or energy distribution for different particle configurations. In this model, the incident plane-wave laser interacts with both SAM-coated and uncoated iron nanoparticles, and the color contour represents the spatial distribution of the electromagnetic field intensity (V/m). The figure visualizes localized surface plasmon resonance (LSPR) and Mie scattering phenomena, where the electromagnetic field becomes strongly concentrated near the nanoparticle surface due to light–matter coupling. The SAM-coated nanoparticle displays a more uniform and symmetric field distribution, indicating enhanced dielectric confinement and reduced field fluctuation caused by the organic monolayer. The SAM layer coating effect stabilizes the resonance condition and promotes consistent field amplification across a broader wavelength range.

In contrast, the uncoated nanoparticle exhibits irregular and asymmetric field localization, reflecting a higher sensitivity to incident wavelength and orientation. The visual evidence of how SAM functionalization modulates near-field enhancement and scattering behavior can be analyzed by confirming that surface engineering plays a decisive role in optimizing nanoparticle-based optical sensors for stable and wavelength-independent electromagnetic responses.

As shown in Figure 4, the figure illustrates the electric field response (V/m) around two different nanoparticle configurations subjected to illumination at visible range spectrum. The upper row (a) represents nanoparticles coated with a 30 nm-thick self-assembled monolayer (SAM), while the lower row (b) shows uncoated nanoparticles (without SAM). In the SAM-coated nanoparticles, the electric field responses show significantly increased intensities compared to uncoated particles. The highest intensities observed in this scenario are limited to around 60 V/m. This subdued electric field distribution indicates that the 30 nm-thick SAM coating effectively enhances electromagnetic interactions, increasing localized field enhancement. Moreover, the distribution patterns demonstrate consistent, symmetric responses across all wavelengths, suggesting that the SAM coating provides stable electromagnetic behavior and effectively moderates the nanoparticle’s response regardless of changes in excitation wavelength.

Conversely, the bottom row depicts the uncoated nanoparticles exhibiting substantially lower maximum electric field intensities, reaching up to approximately 7 V/m. The field enhancement around these particles is visibly weaker and more concentrated, indicating lowered electromagnetic interactions directly at the particle surface. Furthermore, the intensity patterns vary slightly with wavelength, particularly noticeable at shorter wavelengths (400 nm and 500 nm), where a slightly broader and more intense localized field is observed.

Figure 5 illustrates the electric field responses (V/m) of the nanoparticles under infrared (IR) illumination, at wavelengths ranging from 1000 nm to 2500 nm. Similar to previous observations, nanoparticles are presented in two configurations: nanoparticles coated with a 30 nm-thick self-assembled monolayer (SAM) in row (a), and nanoparticles without SAM coating in row (b). Electric field intensities are color-coded, with red representing higher intensities and blue indicating lower intensities.

As shown in Figure 5a, the intensity by the IR laser is significantly lower compared to that of the visible range. The spatial distribution of the electric field is consistently symmetrical, localized near the particle surface, and gradually dissipates outward. This confirms that the SAM coating consistently attenuates the electric field, minimizing wavelength-dependent variations, thus ensuring stable electromagnetic behavior even within the infrared region.

In Figure 5b, the localized electromagnetic field appears stronger, particularly around the particle surface, though still markedly lower in intensity compared to responses observed at visible wavelengths. Moreover, the spatial distribution patterns in this IR range are uniformly symmetric and evenly dispersed, showing less pronounced variability compared to visible wavelengths. Despite this relative stability, uncoated particles still exhibit slightly more dynamic and stronger electromagnetic responses than their SAM-coated counterparts.

Overall, the results emphasize that SAM-coated nanoparticles display stable, consistently reduced electromagnetic interactions under IR illumination. The SAM layer effectively moderates the electric field distribution, offering predictable electromagnetic characteristics valuable for applications requiring controlled, wavelength independent nanoparticle behavior. Conversely, uncoated nanoparticles maintain higher electromagnetic interactions, suggesting potential usefulness in applications that benefit from higher field intensities within the IR region.

**Figure 6:**
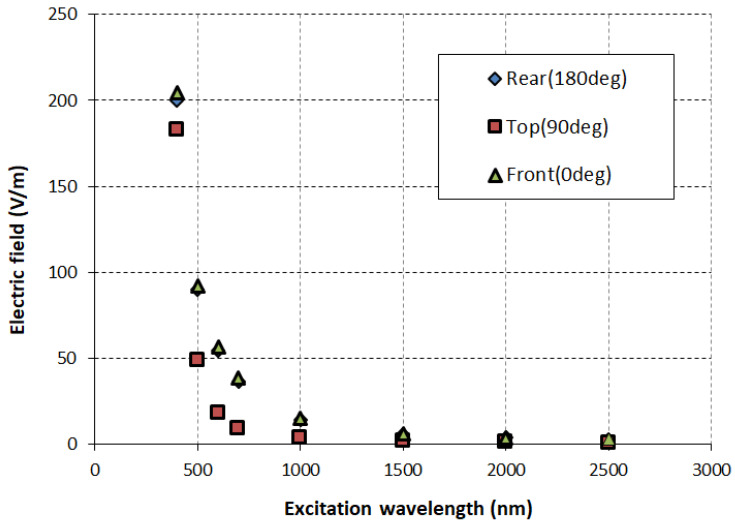
Electric field intensity at different locations for nanoparticles with an SAM.

**Figure 7:**
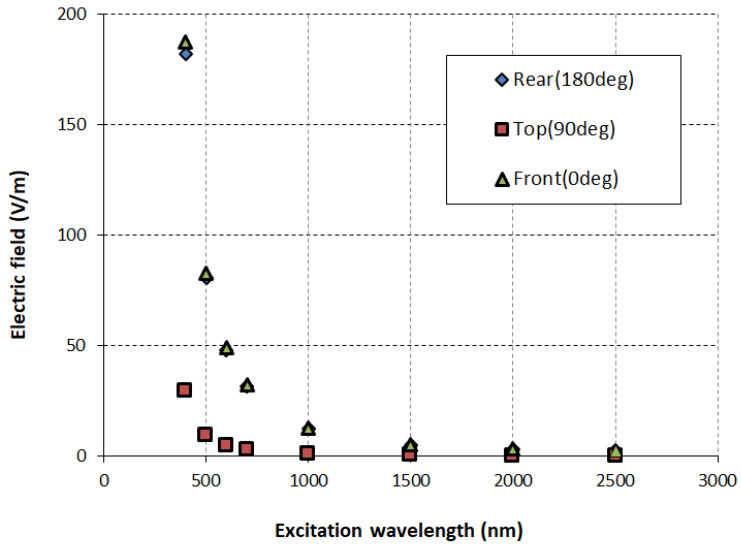
Electric field intensity at different locations for nanoparticles without an SAM.

Figure 6 and Figure 7 shows the electric field enhancement for visible and IR range suggests strong interaction between the incoming EM wave and the iron nanoparticle, with the particle strongly enhancing the electric field on the illuminated side at shorter wavelengths. This enhanced electric field at shorter wavelengths indicates possible resonance phenomena, such as surface plasmon resonance or localized field enhancements due to size-dependent optical effects.

**Figure 8:**
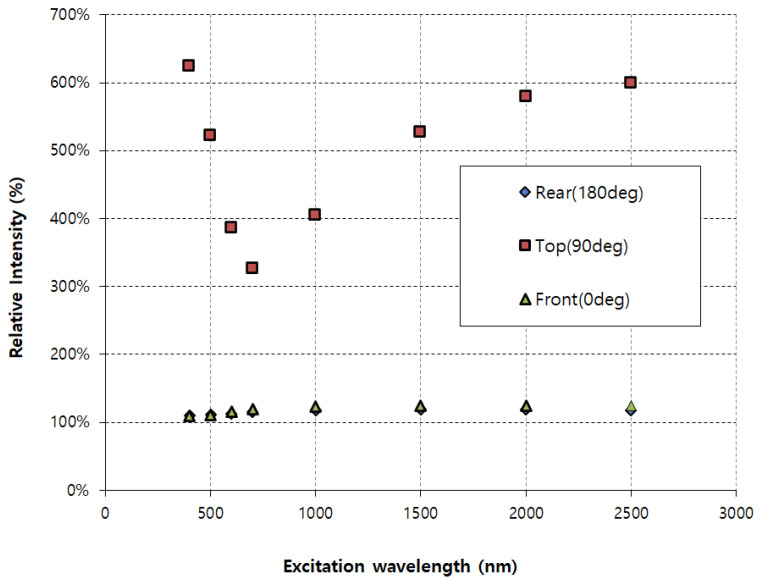
Comparison of electric field intensity.

Figure 8 shows the relative intensity (Ecollioid/Eparticle) at three different detection angles—rear (180°), top (90°), and front (0°)—across a broad wavelength range (approximately 300 nm to 2500 nm). The relative intensity, expressed as a percentage, illustrates how strongly electromagnetic fields are enhanced or attenuated by nanoparticle colloids compared to single particles at different viewing angles. The data reveal that the top angle (90°, red squares) demonstrates significant field enhancement, varying roughly between 350% and 650% across the measured wavelengths, with peak intensity at shorter wavelengths (400–500 nm). Conversely, observations from both front (0°, green triangles) and rear (180°, blue diamonds) angles exhibit much lower and more stable relative intensities, approximately 100–120%, indicating minimal enhancement across all wavelengths. This implies strong angular dependence, with maximum electromagnetic enhancement specifically detected perpendicularly (90°) to the particle alignment. Such information is crucial for optical applications where directional electromagnetic enhancement must be optimized.

## 5. Conclusions

This work highlights how SAM-coated nanoparticles enhance localized electromagnetic fields under laser excitation, providing insights for designing stable and wavelength-independent nanostructures for optical gas sensing. SAM-coated nanoparticles exhibited notable electromagnetic field enhancement compared to their uncoated counterparts in the visible range, with intensities reaching around 60 V/m, demonstrating the capability of SAM layers to significantly amplify localized electromagnetic fields. Moreover, the SAM coatings stabilized and homogenized electromagnetic responses across different wavelengths, providing predictable and consistent field behavior that is beneficial for applications requiring controlled optical interactions, such as targeted drug delivery and diagnostic imaging. In brief, the SAM layer provides chemically active sites that selectively adsorb or interact with gas molecules. The enhanced local electromagnetic field generated under laser excitation increases the excitation rate of molecular vibrational modes or dipole transitions of the adsorbed species, thereby amplifying Raman or infrared absorption intensities in proportion to |E|^4^. This coupling between localized field enhancement and molecular response underlies the improved sensitivity predicted for SAM-functionalized nanoparticles. Replacing Fe with γ-Fe_2_O_3_ or Fe_3_O_4_ (lower |ε|, smaller losses) is expected to reduce the absolute near-field amplitude while preserving the SAM-induced field homogenization and 90° angular enhancement, so the design guidance remains applicable.

The research emphasizes the angular dependence of the electromagnetic field intensity, with maximal enhancement at a perpendicular viewing angle (90°). This angular sensitivity underscores the importance of nanoparticle orientation and detector positioning in practical optical and biomedical applications. In the infrared range, although overall intensity was lower compared to the visible range, SAM coatings effectively moderated the field intensity, providing stable, wavelength-independent electromagnetic characteristics. These insights collectively reinforce the importance of using SAM coatings for enhancing and precisely controlling nanoparticle interactions in diverse technological and biomedical fields.

Simulation-based analysis shows that incorporating the wavelength-dependent optical properties of SAM coatings would further improve the quantitative accuracy of the predicted field enhancements and scattering responses. In the present work, however, the focus is placed on demonstrating that, at a given wavelength, the relative scattering behavior of SAM-coated versus uncoated Fe nanoparticles can be used to distinguish the presence of a surface layer, thereby indicating the feasibility of laser beam scattering as a diagnostic for particle surface state. Future studies will extend this framework by explicitly implementing wavelength-dependent complex refractive indices for various SAM materials, enabling more precise simulations and a broader range of application-specific sensor designs.

Test calculations with wavelength-dependent SAM properties indicate only minor quantitative changes, supporting the use of an effective weakly dispersive SAM model for the present comparative analysis. These results suggest that SAM layers not only supply chemical selectivity but also create an optically enhanced sensing region where gas-induced changes can be more reliably detected via angle-resolved laser scattering.

## Figures and Tables

**Table 1:** Optical properties of the material.

Material	Description	Refractive Index (@550 nm)	Relative Permittivity
Fe (Iron)	Metallic nanoparticle core	2.85 + 3.15i	wavelength-dependent
SAM layer	Organic dielectric coating	1.45	wavelength-dependent (ε_r_ = 2.1 for simulation)
Medium	Air	1.0003	—

## Data Availability

The original contributions presented in this study are included in the article. Further inquiries can be directed to the corresponding author.
